# A Pipeline for ADNI Resting-State Functional MRI Processing and Quality Control

**DOI:** 10.21203/rs.3.rs-11132707/v1

**Published:** 2026-10-06

**Authors:** Saige Rutherford, Zeshawn Zahid, Robert C. Welsh, Andrea Avena-Koenigsberger, Dustin B. Hammers, Vincent Koppelmans, Amanda F. Mejia, Alzheimer’s Disease Neuroimaging Initiative

**Affiliations:** Indiana University; Indiana University; University of California, Los Angeles; Indiana University School of Medicine; Indiana University School of Medicine; University of Utah; Indiana University; University of Southern California

**Keywords:** resting-state fMRI, ADNI, quality control, BIDS, fMRIPrep, reproducibility

## Abstract

The Alzheimer’s Disease Neuroimaging Initiative (ADNI) provides a comprehensive multimodal neuroimaging resource for studying aging and Alzheimer’s disease (AD). However, its resting-state functional MRI (rs-fMRI) data remain underutilized, in part due to challenges with data processing driven by considerable variability in acquisition protocols, inconsistent organization, missing or incomplete imaging sessions, and frequency of data quality issues due to participant motion and aging-related factors. To facilitate widespread use of ADNI rs-fMRI data, we developed an open-source pipeline encompassing the download and organization of imaging and clinical data, conversion to Brain Imaging Data Structure (BIDS), preprocessing with fMRIPrep, and automated quality control (QC). Our QC considers acquisition parameters and 16 structural and functional MRIQC image-quality metrics, flagging scans that fall outside acceptable ranges based on expert input and robust outlier detection techniques. Processed imaging sessions are matched to temporally aligned clinical assessments to produce an analysis-ready dataset. Applying the pipeline to more than 2,800 sessions from ADNI GO, ADNI 2, and ADNI 3 yielded 2,086 quality-checked functional and structural MRI sessions from 935 unique participants. Exclusions occurred at every processing stage and were driven primarily by BIDS conversion errors, image quality flags, and preprocessing- or motion-related failures. The open-source pipeline described in this work produces one of the largest pre-processed, quality controlled, and analysis-ready functional and structural MRI datasets yet available for studying aging and AD. It lowers barriers to large-cohort and longitudinal neuroimaging research and supports large-scale neuroimaging biomarker discovery in AD.

## 1 Introduction

The Alzheimer’s Disease Neuroimaging Initiative (ADNI) is a large multicenter consortium spanning more than 60 academic and clinical sites across the United States and Canada. ADNI collects longitudinal MRI, PET, clinical, cognitive, and biochemical data from cognitively unimpaired older adults, individuals with mild cognitive impairment (MCI), and patients with Alzheimer’s disease (AD) (Weiner et al., 2025), with the goal of standardizing biomarkers and outcome measures for AD clinical trials and developing an accessible longitudinal data repository (Petersen et al., 2010). ADNI has collected data continuously since 2004 across several phases (Table 1).

ADNI has been instrumental in advancing understanding of neurodegeneration by providing large-scale, longitudinal neuroimaging and biomarker data across the spectrum of cognitive aging and dementia (Weiner et al., 2013, 2015, 2024; Yao et al., 2017), with more than 6,000 publications, 30,000 data use agreements, and 405 million image downloads to date (Toga et al., 2024), including 752 papers in 2025 alone. In contrast, relatively few studies have leveraged ADNI resting-state fMRI (rs-fMRI): a targeted PubMed search (full search strategy in Supplementary Material) identified only 352 publications using ADNI rs-fMRI as of 2025 (Fig. 1A), just 5.9% of all ADNI publications. In 2025 alone, only 48 of 752 ADNI papers (6.4%) used rs-fMRI.

This gap is not fully explained by the later start of rs-fMRI acquisition (ADNI-GO in 2009 vs. ADNI-1 in 2004); instead, it likely reflects structural barriers to reuse rather than a lack of scientific interest. Rs-fMRI data are inherently more complex, require more extensive preprocessing, and are sensitive to acquisition and quality-control variability, factors reflected in the wide variation in published sample sizes, from 2 to 1326 subjects (Fig. 1B). This underutilization represents a major opportunity: ADNI rs-fMRI is a rich, longitudinal, yet largely untapped resource for studying functional brain changes in aging and AD, one that modern data standards and processing tools are positioned to unlock.

A reproducible, transparent, semi-automated preprocessing pipeline for ADNI functional and structural MRI data will accelerate large-scale secondary analyses of this resource and provide the transparent documentation that reproducible neuroimaging research requires (Gau et al., 2021; Nichols et al., 2017). Here we describe a pipeline that converts ADNI functional and structural MRI data into the Brain Imaging Data Structure (Gorgolewski et al., 2017), applies standardized preprocessing, performs automated quality assessment, flags data requiring troubleshooting, and integrates clinical assessments. The pipeline combines established tools including Clinica (Routier et al., 2021; Samper-González et al., 2018), fMRIPrep (Esteban, Markiewicz, et al., 2018), and MRIQC (Esteban et al., 2017), with bespoke quality control tools and an optional containerized environment for reproducibility across computing systems. Our configuration files and processing scripts provide a complete blueprint for producing an analysis-ready dataset within the constraints of the ADNI data use agreement, supporting functional biomarker discovery in AD and related neurodegenerative conditions.

## 2 Methods

Here, we describe a complete data curation pipeline for ADNI functional and structural MRI, organized according to nine main workflow steps (Fig. 2) implemented in an public GitHub repository (https://github.com/statMINDlab/ADNI_fMRI_protocol). For each step, we specify the data inputs, processing operations, software tools and versions, quality control procedures, and known issues specific to ADNI. All scripts and supporting files referenced below are available in the repository. This pipeline currently supports the ADNI GO, ADNI 2, and ADNI 3 datasets.

Data used in the preparation of this article were obtained from the ADNI database (adni.loni.usc.edu). ADNI was launched in 2003 as a public-private partnership, led by Principal Investigator Michael W. Weiner, MD. The primary goal of ADNI has been to test whether serial MRI, PET, other biological markers, and clinical and neuropsychological assessment can be combined to measure the progression of mild cognitive impairment (MCI) and early AD. For up-to-date information, see https://adni.loni.usc.edu/.

### Step 1: Account Setup and Data Use Agreement

Raw imaging and clinical data are accessed through the Laboratory of Neuro Imaging Image and Data Archive (LONI/IDA) (https://ida.loni.usc.edu/) after account registration and approval of ADNI’s Data Use Agreement (DUA). The protocols here provide detailed instructions (further detailed instructions are available in the folder s1_setup_account/) for users to reproduce the entire workflow independently; however, no raw data are shared via our repository, in compliance with ADNI’s DUA. All processing is performed within secure institutional or high-performance computing (HPC) environments, consistent with ADNI DUA requirements.

### Step 2: Data Selection and Download from LONI

The second step involves selecting eligible ADNI sessions and downloading the associated imaging files, metadata spreadsheets, and study information packages (further instructions in s2_download/). Using the “Image Collections” tool in LONI/IDA, users can filter for resting-state fMRI sequences and associated structural sequences (T1 & T2) across the ADNI GO, ADNI 2, and ADNI 3 phases. ADNI 1 is excluded because it does not include resting-state fMRI, and Clinica does not yet support ADNI 4. The GitHub repository for the pipeline includes step-by-step screenshots and instructions for building reproducible LONI queries. All downloaded ZIP files, including raw imaging archives (DICOM format), metadata spreadsheets (e.g., ADNIMERGE.csv), and ADNI study files (e.g., clinical/behavioral data), should be stored as defined in the user’s configuration file. For the current manuscript, data released prior to October 1st, 2025, were downloaded and used for the development and testing of the pipeline.

### Step 3: DICOM Organization and Integrity Checks

After download, all ZIP archives are uncompressed, reorganized into a standardized directory structure, and subjected to basic data quality checks. Scripts in s3_organize/verify the DICOM download (expected number of directories based on image collection) and map the heterogeneous DICOM directory names to their corresponding imaging modalities (i.e., T1, T2, fMRI (i.e., BOLD)).

### Step 4: Imaging and Clinical Data Conversion Using Clinica

Clinica is an open-source software platform that provides standardized converters from raw acquisition formats (e.g., ADNI DICOMs) to the Brain Imaging Data Structure (BIDS). DICOM files are converted into BIDS format (v1.10.1) (Gorgolewski et al., 2017) format using Clinica (v0.10.1) (Routier et al., 2021; Samper-González et al., 2018). The command-line interface and configuration parameters are provided in s4_clinica/. Clinica serves two main functions: i) DICOM to NIfTI conversion and metadata extraction (via dcm2niix) and ii) organizing data into BIDS format. Clinica cross-references session dates using several ADNI spreadsheets (e.g., ADNIMERGE, MPRAGEMETA, and quality control tables) to ensure accurate mapping of T1, T2, and BOLD series to visit codes. After conversion, the resulting dataset is validated using the BIDS Validator (v1.14.7). Each participant’s BIDS dataset contains both structural (T1 and, if available, T2) and functional (BOLD) images, along with all required metadata fields necessary for downstream processing with fMRIPrep and MRIQC.

During the development of this pipeline, we identified several issues in the ADNI DICOMs that must be addressed prior to preprocessing. These include items flagged by Clinica (interslice distance variation and failed image conversion, both documented in s4_clinica/known_clinica_DICOM_errors.csv) and several problems we encountered that were not reported by Clinica (missing or incorrect PhaseEncodingDirection parameter, missing JSON sidecar files for T1 files, T1 DICOM conversion errors, and absent SliceTiming metadata for BOLD series acquired on Philips scanners). Our scripts automatically flag these issues. Subjects with unrecoverable errors are excluded at this stage. For a more detailed description of Clinica, including some of the decisions about which scan to select (e.g., if there are multiple T1 per session), we refer readers to their detailed documentation (https://aramislab.paris.inria.fr/clinica/docs/public/dev/Converters/ADNI2BIDS/) and user support Google group (https://groups.google.com/g/clinica-user).

### Step 5: Post-Clinica Metadata Correction and Data Quality Checks

Philips BOLD scans lack the slice-acquisition timing metadata needed for slice timing correction during preprocessing with fMRIPrep. As such, Philips scans would be processed differently from scans acquired on Siemens and GE systems. To ensure consistent preprocessing across scanner manufacturers, we reconstruct the slice-acquisition timing for Philips scans using information available in tables downloaded from LONI (MAYOADIRL_MRI_FMRI_NFQ_*.csv). We then add it to the BOLD metadata prior to preprocessing to ensure slice timing correction is performed correctly. Scans for which slice timing cannot be determined unambiguously are flagged and excluded.

After DICOM and BIDS conversion, additional QC steps ensure that each session meets minimum functional and structural imaging criteria (s5_post_clinica_qc/). Five heuristics regarding the scanner parameters are applied (Table 2). These heuristics were determined by an expert in MR physics (author RCW) and were chosen to detect errors in the data that occurred during collection. All scanner parameters were reviewed across sites/scanners to aid in the heuristic threshold choices. A notebook (s5_post_clinica_qc/analysis/create_report/main.ipynb, also available as a Python script) creates per-site and per-manufacturer (i.e., Siemens, GE, Philips) QC plots, as well as the summary tables of scanner parameters and inclusion/exclusion sample sizes at each decision point. It also summarizes and categorizes Clinica output errors (e.g., missing or incorrect *PhaseEncodingDirection*, missing JSON sidecar files for T1 files, or T1 DICOM conversion errors). The final step of this notebook is to export a CSV file with the subjects and sessions to be passed on to MRIQC and fMRIPrep.

### Step 6: Automated QC with MRIQC

MRIQC is an open-source, automated quality control tool that computes standardized image quality metrics (IQMs) directly from T1-weighted and BOLD MRI data, without requiring a reference dataset (Esteban et al., 2017). Sixteen automated participant-level IQMs are generated by MRIQC (version 24.0.2) for each individual session, and group-level distributions are used to determine quantitative exclusion criteria. The full list of IQMs is provided in Table 3.

There are no established thresholds for these IQMs, to our knowledge. To determine cutoffs for these IQMs in an automated fashion, we use a robust statistical outlier detection method developed by our lab (Parlak et al., 2025). For each IQM, the distribution across sessions is transformed to approximately follow a Normal distribution using a statistically robust, flexible transformation to eliminate skew and heavy or light tails. In the standard Normal distribution, values beyond ± 4 are extremely unlikely (probability < 0.000063). Thus, after transformation, values above 4 or below − 4 (depending on whether higher or lower values of the IQM are considered better, see Table 3) are flagged. These cutoffs are translated back to the original scale of the data by applying the reverse transformation. The code implementing this procedure and for producing visualizations is located in s6_mriqc/.

### Step 7: fMRIPrep Preprocessing

Image preprocessing is performed using fMRIPrep version 25.2.3 (Esteban, Blair, et al., 2018), which includes surface reconstruction and projection via Freesurfer. For more details of the fMRIPrep pipeline, see fMRIPrep‘s workflows documentation (https://fmriprep.org/en/stable/) and citation boilerplate. The scripts for this step and further instructions are in the directory s7_fmriprep/.

We systematically parsed fMRIPrep logs to categorize failures (missing BOLD files, TemplateFlow cache/bind issues, insufficient memory, FreeSurfer recon-all failures, and fieldmap-less distortion correction failures). We designed upstream checks and conditional execution logic, which ensure that jobs are only submitted when all required inputs are present. Full details of each failure mode, and how they are resolved, are provided in the Supplementary Material.

### Step 8: Final QC and Derivation of the Analysis Sample

The final QC stage comprises quality checking the subjects and sessions that successfully completed fMRIPrep and all previous steps. This includes summarizing the quality of the Freesurfer surface reconstruction and checking the functional runs for motion. Surface reconstruction quality is checked using the Euler number, a measure of the topological complexity of the surface, since higher topological complexity is considered to be indicative of problems with surface reconstruction. It has been shown to correlate highly with manual quality checking ratings (Rosen et al., 2018) and is the main metric used in automated QC methods (Klapwijk et al., 2019). Since the distribution of the Euler number varies across sites, we adopt a simple approach that involves scaling and centering the distribution within site and removing samples in the tail of the distribution (Kia et al., 2021). After censoring based on a framewise displacement (FD) threshold of 0.5 mm, sessions with more than 30% of frames censored or less than 5 minutes of low-motion data remaining are excluded. These thresholds can be adjusted by users as desired. A consolidated inclusion/exclusion table (with reasons coded per session) is produced in s8_final_qc/. The final analysis-ready dataset includes all subjects meeting QC criteria across every stage of this protocol (DICOM download, Clinica DICOM-to-NIfTI conversion and BIDS formatting, MRIQC, and fMRIPrep preprocessing).

### Step 9: Merging of clinical and imaging visits

The final step of the pipeline involves matching imaging sessions to clinical assessments for each participant. Because imaging and clinical assessments are not always collected on the same calendar date, each BIDS session is paired with the clinical visit whose assessment date falls closest in time, subject to a maximum allowable gap. The default maximum is 180 days, but this can be adjusted by the user via the configuration file. Sessions without a clinical visit within this window are flagged, rather than silently dropped.

Session dates are extracted during BIDS conversion (Section 2.4), and visit codes (VISCODEs) are obtained from ADNIMERGE. This is not simple renaming: ADNI VISCODEs (e.g., m06, m12) do not always correspond one-to-one with imaging visit numbers, so the matching is performed explicitly on visit dates, rather than assumed from VISCODE labels alone. The resulting merged table links each participant_id/session_id pair to its aligned clinical, cognitive, and diagnostic variables (including diagnosis at that visit, MMSE, ADAS, MOCA, and CDR scores) and is written to clinical_data.tsv. Other behavioral and clinical variables can be included if desired, with minor modifications to the code. Scripts implementing this matching procedure are provided in s9_clinical_imaging_merge/.

## 3 Results

Applying this protocol to ADNI GO, ADNI 2, and ADNI 3 (data downloaded through October 1, 2025) yields a final analysis-ready dataset of 935 participants and 2,086 rs-fMRI sessions. Table 4 reports the number of subjects and sessions retained and dropped at each stage of the pipeline, from the initial download (1,153 subjects, 2,893 sessions) to final inclusion, based on our ADNI data download. This illustrates the cumulative effects of download issues, metadata inconsistencies, BIDS conversion failures, MRIQC-based outlier detection, fMRIPrep preprocessing errors, and final motion/Euler-number quality thresholds on the resulting analysis sample. Documentation for producing a finalized sample-size summary (e.g., Table 4 and Fig. 4) directly from the pipeline outputs is provided in the Supplementary Material and the accompanying GitHub repository (see code availability below). In the initial QC phases (BIDS-convert via Clinica and Post-Clinica QC), subjects were excluded due to missing data or metadata (28 subjects/210 sessions) or incompatible scanner parameters (described in Table 2) (20 subjects/62 sessions).

As described above (Step 6), automated outlier detection identified structural and functional scans with atypical MRIQC image quality metrics (IQMs). Figure 3 visualizes the distribution of the IQMs for structural and functional data, along with the thresholds for exclusion. A scan was classified as failed if *any* IQM exceeded its threshold. If the T1 image failed, the BOLD image was also excluded since the BOLD cannot be successfully preprocessed without a high-quality T1 image from the same session. A total of 46 sessions were dropped due to missing IQMs (i.e., MRIQC failed, likely due to poor quality data), and 86 sessions were dropped due to IQMs outside of the determined thresholds.

Examples of scans excluded for each metric are shown in the supplement (Supplemental Figs. 1–7). Example scans were selected i) at random and ii) near the exclusion threshold to determine that the automatic metrics were catching true outliers with poor quality data. We also visualize the distribution of each IQM per site in the supplement (Supplemental Figs. 8–9). This approach offers an automated, reproducible, and interpretable strategy for identifying problematic scans in heterogeneous, multicenter datasets.

During fMRIPrep preprocessing, a small number of sessions required reprocessing without fMRIPrep’s fieldmap-less distortion correction (predominantly Philips acquisitions missing *PhaseEncodingDirection* metadata). A total of 375 sessions (121 subjects) were excluded due to unrecoverable FreeSurfer recon-all and fMRIPrep preprocessing failures (Step 7; Supplementary Material). At the final QC stage, 28 sessions with excessive head motion (> 30% censored frames or < 5 minutes of low-motion data) or outlying Euler numbers were removed, yielding the final analysis-ready sample of 2,086 rs-fMRI sessions from 935 participants reported in Table 4.

The resulting sample spans the diagnostic spectrum captured by ADNI (cognitively unimpaired, MCI, and AD/dementia) and all three scanner manufacturers (Siemens, GE, and Philips) represented across ADNI GO/2/3 sites. The distribution and summary statistics of imaging-to-clinical-visit time gaps, as well as an overview of all longitudinal sessions, are visualized in Fig. 4.

## 4 Discussion

Resting-state fMRI provides a non-invasive window into large-scale functional brain organization and has shown promise for detecting early network-level changes associated with cognitive decline and Alzheimer’s disease (Forouzannezhad et al., 2019; Vemuri et al., 2012). Furthermore, ADNI is a richly deep and unique dataset whose impact continues to grow. Even so, ADNI rs-fMRI data have remained substantially underutilized relative to structural and PET imaging, largely due to the complexity of data curation, preprocessing, and quality control in a heterogeneous, multisite dataset.

In this work, we describe a data curation pipeline that addresses these challenges by providing a framework for transforming raw ADNI rs-fMRI data into an analysis-ready dataset linked to clinical assessments. The pipeline integrates clinical-imaging data alignment, standardized preprocessing, and multi-level quality control, with decisions at each stage documented and automated to support reproducibility. The pipeline lowers the technical barriers to using ADNI functional MRI data at scale. This is particularly important as research employing rs-fMRI increasingly moves toward large cohorts suitable for biomarker discovery, longitudinal modeling, and machine learning. This work enables researchers to leverage ADNI as a large longitudinal rs-fMRI resource for studying aging and AD. The resulting dataset facilitates the study of functional connectivity changes across disease stages, evaluation of fMRI-based prognostic markers, and integration of functional measures with the structural, PET, and fluid biomarkers already widely used in ADNI-based research.

Several limitations of the existing pipeline are worth noting. First, the pipeline relies on external tools, including Clinica, MRIQC, and fMRIPrep, and therefore inherits any limitations, issues, or design decisions associated with those tools. For instance, Clinica, which performs the essential step of conversion of ADNI data to BIDS format, does not currently support ADNI-4 data; accordingly, our pipeline currently covers through ADNI-3 only and will require corresponding upstream support to incorporate ADNI-4. Additionally, some of Clinica’s data selection rules are not fully configurable by users, and its limited version control may inhibit full reproducibility. Several additional limitations arise from our own design decisions. For instance, while our quality control heuristics applied at Step 5 reflect expert judgment regarding plausible scan acquisition parameter ranges, the MRIQC IQM thresholds applied at Step 6 are based on an automated outlier detection technique using transformation to a normal distribution; these thresholds are not theoretically motivated, have not been validated against the stability of derived connectivity estimates, and depend on the quality of the normality transformation. IQMs may also benefit from site-specific thresholding, whereas we applied a universal threshold. Additionally, the 180-day window used to match clinical assessments to imaging sessions is generous relative to the observed distribution of intervals, and analyses treating cognitive scores as concurrent with imaging may warrant a more conservative threshold. Importantly, however, the pipeline allows investigators to adjust QC thresholds, window length, and other design decisions to suit the requirements of a given analysis and to assess the sensitivity of their results to these choices.

A related issue concerns the T1-weighted images available for a subset of ADNI sessions. In some cases, the only T1 image ADNI provides for a given session has already undergone processing (e.g., gradient-nonlinearity correction or intensity normalization) rather than representing the raw acquisition, and this processing history is not consistently documented in the accompanying metadata. Because Clinica selects among the T1 series that ADNI makes available, it cannot always distinguish a processed image from a raw one when ADNI itself does not flag the distinction. We therefore view this as a limitation of ADNI‘s metadata and provenance documentation rather than of Clinica specifically, and it highlights a broader need for more complete and standardized provenance tracking across the ADNI archive.

Future work will focus on extending this resource in ways that support the broader goals of aging and AD research. A priority is incorporating ADNI-4 data as upstream tool support becomes available, so that the pipeline continues to grow alongside the ADNI archive itself. We also plan to pursue harmonization strategies that reduce site- and scanner-related variability, making it easier to pool this resource with other multicenter datasets. Finally, we see promise in using large, curated resources like this one to inform individualized brain mapping, for instance by contributing to Bayesian priors that improve the precision of person-specific estimates of brain structure and function. Together, these directions aim to make ADNI‘s functional imaging data an increasingly powerful resource for understanding aging and Alzheimer‘s disease.

In summary, we present a complete and open pipeline for transforming ADNI resting-state fMRI data into an analysis-ready resource. Applied to the full ADNI archive through ADNI-3, the pipeline yielded 935 participants and 2,086 sessions with curated and processed rs-fMRI data, verified scanner parameters and quality controls, and clinical assessments matched to each imaging session. A key strength of this work is the transparency and flexibility of the overall workflow: curation, preprocessing, and quality control decisions are documented and reproducible, while thresholds and other design choices can be adjusted to meet the needs of a given analysis. By facilitating more widespread and large-scale use of the ADNI resting-state fMRI data, this resource will support studies of functional brain changes and biomarker development in aging and Alzheimer’s disease. More broadly, this work provides a practical framework for bringing legacy neuroimaging datasets into contemporary, reproducible research workflows to accelerate reuse of existing neuroimaging resources.

## Supplementary Material

Supplementary Files

This is a list of supplementary files associated with this preprint. Click to download.

- SupplementaryMaterial.docx

## Figures and Tables

**Figure 1:**
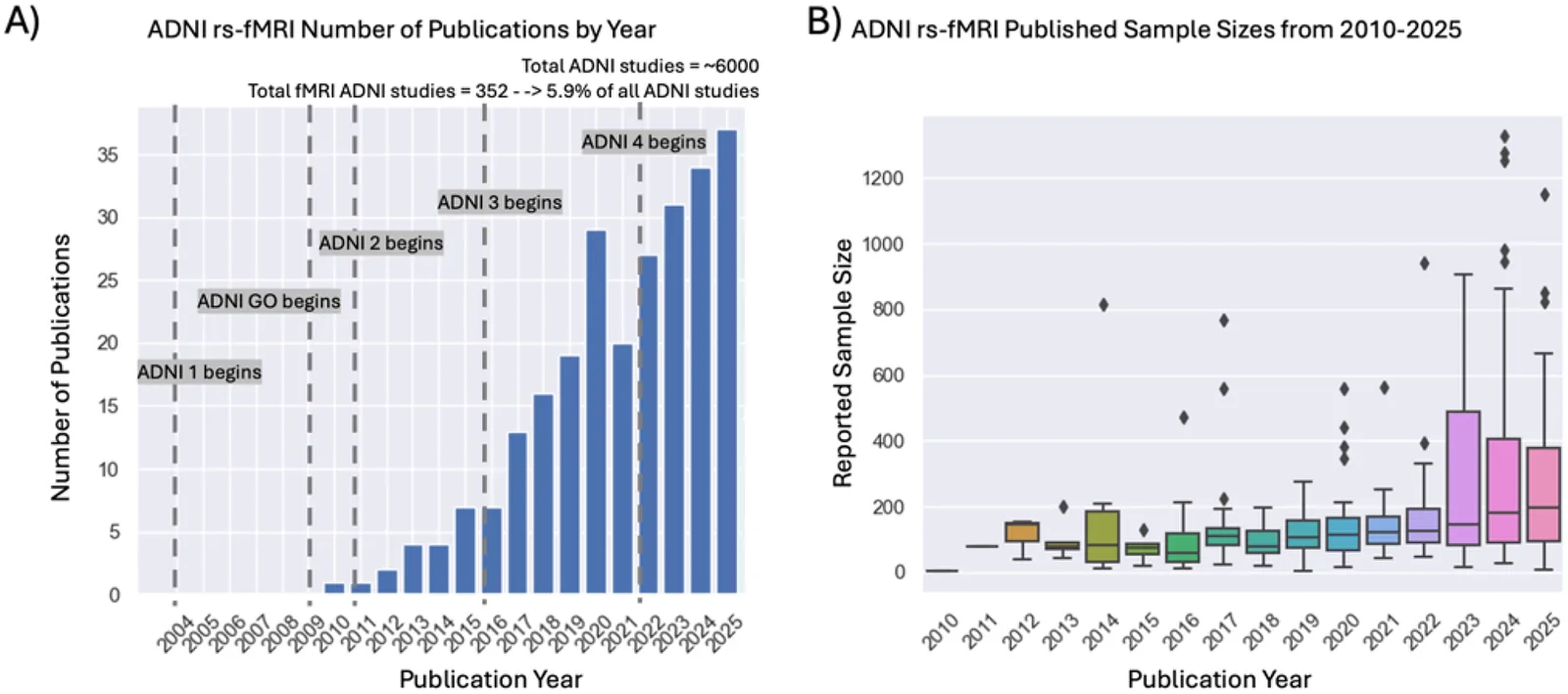
**A)** The number of published papers using ADNI rs-fMRI data per year (2010–2025). A full list of these papers is provided in the supplement (Supplemental File 1). **B)** The reported sample sizes in each publication using ADNI rs-fMRI data, per year (2010–2025). The average sample size across all publications is 195 subjects (SD=222).

**Figure 2:**
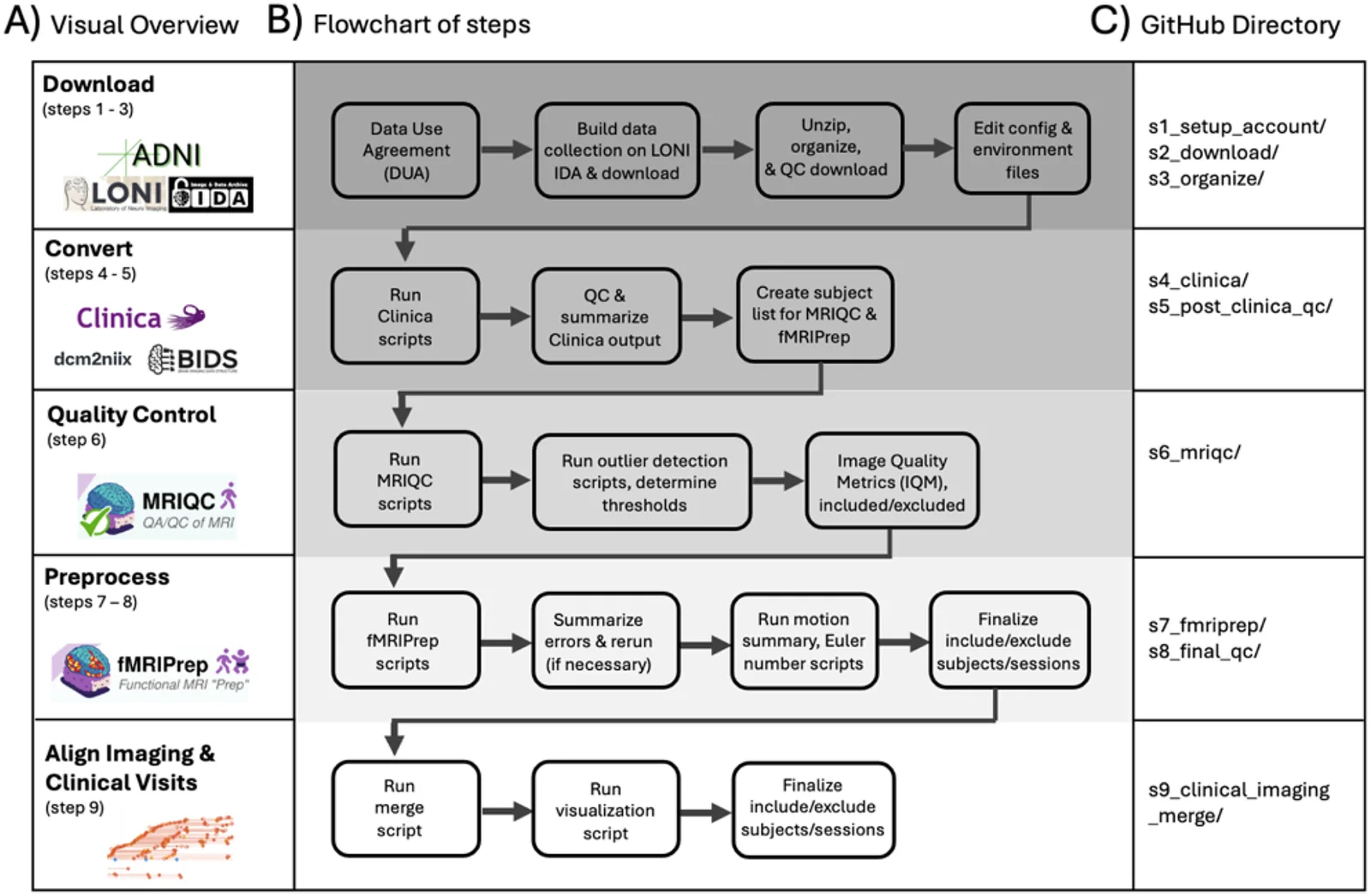
Overview of the nine steps in the ADNI fMRI data curation and processing pipeline. Step numbers (panel A) correspond to the detailed steps in the GitHub repository (panel C) (https://github.com/STATMINDlab/ADNI_fMRI_protocol).

**Figure 3:**
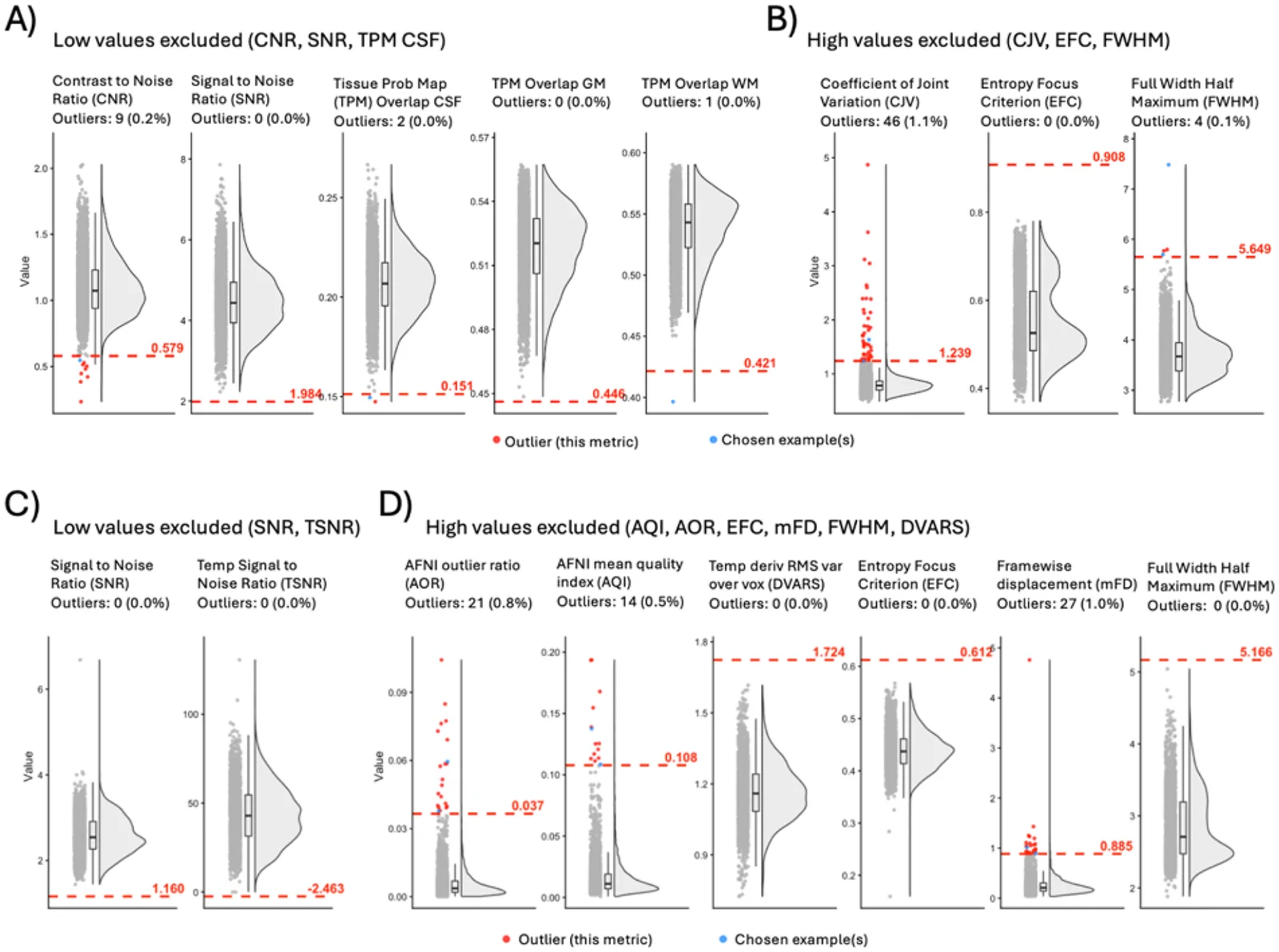
**Top row:** MRIQC Image Quality Metrics (IQMs) for structural (T1) images. These metrics are explained in Table 3. The red line indicates the outlier threshold. **A)** images below this line are excluded, and **B)** images above this line are excluded. Subjects were excluded if they had an outlier on any of these. **Bottom row:** MRIQC Image Quality Metrics (IQMs) for BOLD images. These metrics are explained in Table 3. The red line indicates the outlier threshold. **C)** images below this line are excluded, and **D)** images above this line are excluded. Sessions were excluded if they had an outlier on any of these.

**Figure 4:**
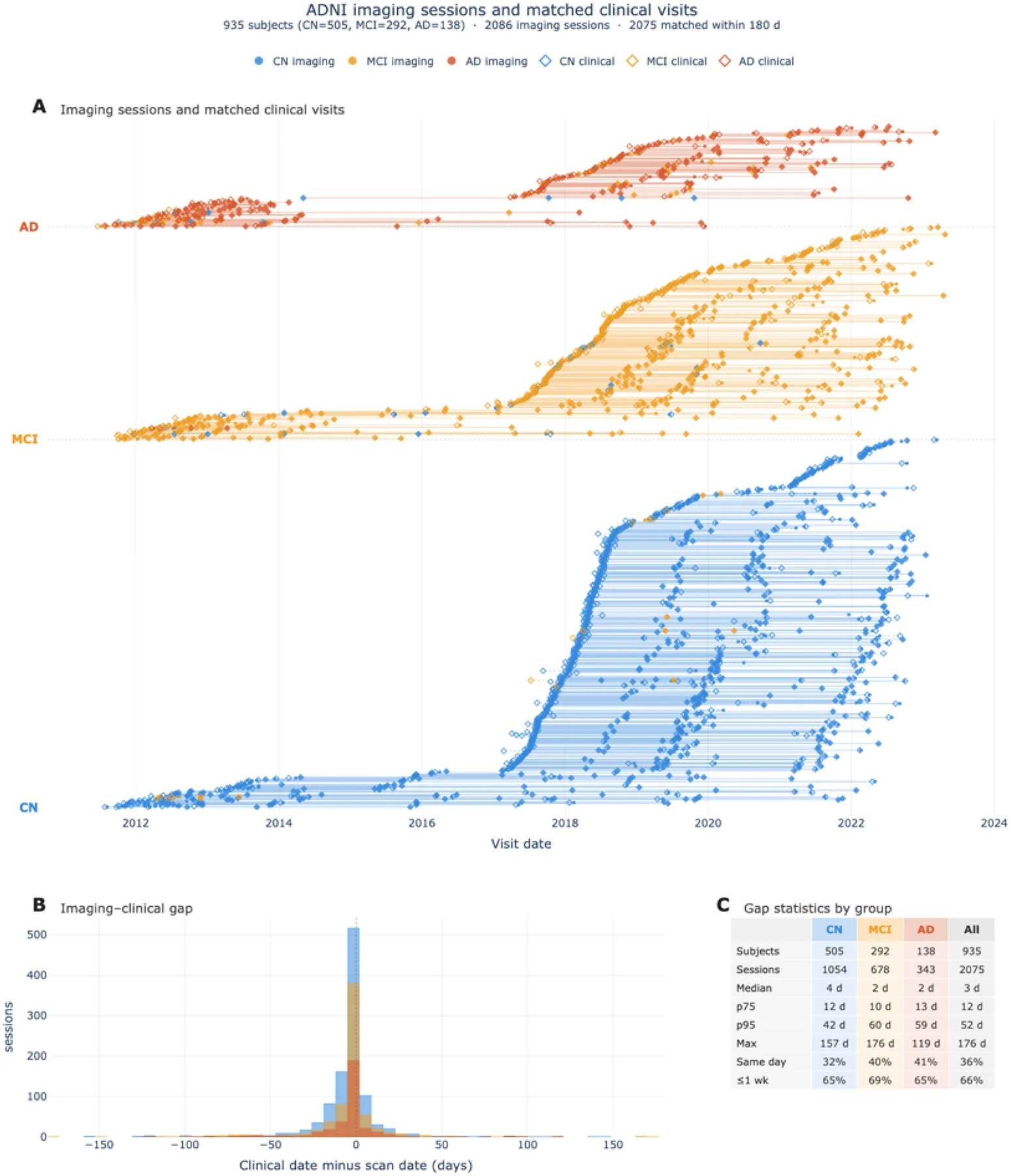
Imaging sessions and matched clinical assessments in the final ADNI rs-fMRI sample. **A)** Visit timelines for all 913 subjects in the final sample, grouped by *final* diagnostic group (CN, MCI, AD). Filled circles denote imaging sessions, colored by diagnosis recorded at that visit; open diamonds denote clinical assessments; dotted segments connect a scan to its matched assessment and are therefore proportional to the interval between them. Solid horizontal lines link successive scans and are colored by final diagnosis. Subjects entering at later study phases appear as a staircase indicating a large temporal gap. **B)** Distribution of the signed interval between matched visits by diagnostic group. **C)** Summary of the absolute clinical-to-scan interval by diagnostic group. Panels B and C exclude 16 sessions whose scan dates were imputed and 12 sessions with no clinical visit within the 180-day matching window.

**Table 1:** Overview of the phases of ADNI and the primary goal, timeline/duration, funding, and cohort description of each phase. Each cohort after ADNI 1 includes the previous cohort(s). This table was recreated from ADNI’s website. CU = Cognitively Unimpaired, MCI = Mild Cognitive Impairment, AD = Alzheimer’s Disease, DEM = Dementia.

Timeline (Duration)	ADNI 1	ADNI GO	ADNI 2	ADNI 3	ADNI 4
	2004–2010	2009–2011	2011–2016	2016–2022	2022–2027
	(5 years)	(2 years)	(5 years)	(5 years)	(5 years)
**Primary Goal**	To develop biomarkers as outcome measures for clinical trials.	Examine biomarkers in earlier stages of disease.	Develop biomarkers as predictors of cognitive decline, and as outcome measures.	Study the use of tau PET and functional imaging techniques in clinical trials.	Improve generalizability of AD research.
**Cohort**	200 CU, 400 MCI, 200 AD	+ 200 early MCI	+ 150 CU, 150 elderly MCI, 150 late MCI, 150 AD	+ 133 CU, 151 MCI, 87 AD	+ 200 CU, 200 MCI, 100 AD/DEM

**Table 2:** Heuristics for excluding subjects/sessions based on scanner parameters.

Metric	Exclusion Rule	Rationale
ScanDepth = dim_3_ × pixdim_3_	Outside range [155, 180]	Ensures adequate brain coverage
Repetition Time (TR)	Outside [0.5–1.0] or [2.9–3.1] seconds	Detects mislabeled scans or protocol inconsistencies
Minimum total scan duration	< 300 seconds	Ensures ≥ 5 minutes of fMRI data
PercentPhaseFOV	< 72	Indicates spatial under sampling
CoilString	Q-Body or BODY coils	Nonstandard or problematic coils

**Table 3:** Automated image quality metrics from MRIQC.

Name	Used for	Description	Direction
Contrast to Noise Ratio (CNR) (Magnotta et al., 2006)	T1	Extension of the SNR to evaluate how separated the tissue distributions of GM and WM are.	Higher = better
Signal to Noise Ratio (SNR) (Kaufman et al., 1989)	T1	The mean intensity within gray matter divided by the standard deviation of the values outside the brain.	Higher = better
Tissue probability overlap map (TPM) of Cerebrospinal Fluid (CSF)	T1	Overlap estimated from the CSF image and the corresponding maps from the ICBM nonlinear-asymmetric 2009c template.	Higher = better
TPM of Gray Matter (GM)	T1	Overlap estimated from the GM image and the corresponding maps from the ICBM nonlinear-asymmetric 2009c template.	Higher = better
TPM of White Matter (WM)	T1	Overlap estimated from the WM image and the corresponding maps from the ICBM nonlinear-asymmetric 2009c template.	Higher = better
Coefficient of Joint Variation (CJV) (Ganzetti et al., 2016)	T1	CJV of GM and WM: objective function for the optimization of INU correction algorithms. Higher values are related to the presence of heavy head motion and large INU artifacts.	Lower = better
Entropy Focus Criterion (EFC) (Atkinson et al., 1997)	T1	Uses the Shannon entropy of voxel intensities as an indication of ghosting and blurring induced by head motion.	Lower = better
Full Width Half Max (FWHM (mm)) (Friedman et al., 2008)	T1	The FWHM of the spatial distribution of the image intensity values in units of voxels.	Lower = better
Signal to Noise Ratio (SNR) (Kaufman et al., 1989)	BOLD	The mean intensity within gray matter divided by the standard deviation of the values outside the brain.	Higher = better
Temporal Signal to Noise Ratio (TSNR)	BOLD	Median value of the tSNR map (average BOLD signal / temporal standard deviation).	Higher = better
AFNI’s mean quality index (AQI) (Cox, 1996)	BOLD	Mean quality index as computed by AFNI’s 3dTqual for each volume, it is one minus the Speaman’s rank correlation of that volume with the median volume.	Lower = better
AFNI’s outlier ratio (AOR) (Cox, 1996)	BOLD	Mean fraction of outliers per fMRI volume as given by AFNI’s 3dToutcount.	Lower = better
Entropy Focus Criterion (EFC) (Atkinson et al., 1997)	BOLD	Uses the Shannon entropy of voxel intensities as an indication of ghosting and blurring induced by head motion.	Lower = better
Framewise displacement (meanFD) (Power et al., 2014)	BOLD	Expresses instantaneous head-motion. Rotational displacements are calculated as the displacement on the surface of a sphere of radius 50 mm.	Lower = better
Full Width Half Max (FWHM (mm)) (Friedman et al., 2008)	BOLD	The FWHM of the spatial distribution of the image intensity values in units of voxels.	Lower = better
Temporal derivative of RMS variance over voxels (DVARS) (Power et al., 2012)	BOLD	Indexes the rate of change of BOLD signal across the entire brain at each frame of data.	Lower = better

**Table 4:** Inclusion and exclusion sample sizes at each step of the protocol. The Dropped column reports how many subjects (sub) and sessions (ses) were excluded from the previous step (row). The final column reports the sample size after exclusions up to each step. The sample consists of data from ADNI-GO, ADNI-2, and ADNI-3, the data releases used in our demonstration of the pipeline.

Pipeline Stage	GitHub Folder	Description	Dropped (Sub/Ses)	Subjects/Sessions
Unzip & organize DICOMs	s3_organize/	Clean directory tree; check integrity	-	1153/2893
BIDS-convert via Clinica	s4_clinica/	NIfTI + JSON + sidecars	28/210	1125/2683
Post-Clinica QC	s5_post_clinica_qc/	TR/coverage/duration checks	20/62	1105/2621
MRIQC	s6_mriqc/	All IQMs present; MRIQC ran successfully	12/46	1093/2575
Post-MRIQC QC	s6_mriqc/	Robust outlier detection	25/86	1068/2489
fMRIPrep	s7_fmriprep/	Volumetric, surface, grayordinates outputs	121/375	947/2114
Final QC	s8_final_qc/	Euler number, motion, ≥ 5 min usable data	12/28	**935/2086**

## Data Availability

All code required to reproduce the complete ADNI resting-state fMRI preprocessing workflow is openly available in the accompanying GitHub repository (https://github.com/STATMINDlab/ADNI_fMRI_protocol). All workflows and scripts are version controlled. A static, citable snapshot of the repository (including the configuration files, QC code, and documentation) will be archived on Zenodo, and a DOI will be provided upon publication. Full details of the repository structure and file contents are provided in the Supplementary Material. The raw and processed ADNI imaging, clinical, and biomarker data analyzed in this study are available to qualified researchers upon approval of a Data Use Agreement through the ADNI data archive (adni.loni.usc.edu). The processed, quality-controlled derivatives generated by this pipeline (BIDS-formatted imaging data, QC tables, and matched clinical assessments) can be regenerated by any researcher with ADNI data access using the code and documentation described above.

## Data Availability

The raw and processed neuroimaging, clinical, and biomarker data analyzed in this study are available from the ADNI data archive (adni.loni.usc.edu) upon approval of a Data Use Agreement. Code required to regenerate all derivatives described in this manuscript is available as described in the Information Sharing Statement above.
